# Transthoracic Echocardiographic Assessment of the Heart in Pregnancy—a position statement on behalf of the British Society of Echocardiography and the United Kingdom Maternal Cardiology Society

**DOI:** 10.1186/s44156-023-00019-8

**Published:** 2023-04-20

**Authors:** Stephanie L. Curtis, Mark Belham, Sadie Bennett, Rachael James, Allan Harkness, Wendy Gamlin, Baskaran Thilaganathan, Veronica Giorgione, Hannah Douglas, Aisling Carroll, Jamie Kitt, Claire Colebourn, Isabel Ribeiro, Sarah Fairbairn, Daniel X. Augustine, Shaun Robinson, Sara A. Thorne

**Affiliations:** 1 University Hospitals Bristol and Weston NHS Trust, Bristol Heart Institute, Marlborough Street, Bristol, BS2 8HW UK; 2 Cambridge University Hospitals NHS Foundation Trust, Cambridge, UK; 3 University Hospitals of North Midlands, Stoke-On-Trent, UK; 4 University Hospitals Sussex NHS FT, Brighton, UK; 5 United Kingdom’s Maternal Cardiology Society, London, UK; 6 East Suffolk and North Essex NHS Foundation Trust, Essex, UK; 7 North West Heart Centre, Wythenshawe Hospital, Manchester, UK; 8 St George’s University Hospitals NHS Trust, London, UK; 9 Guy’s and St Thomas’ NHS Trust, London, UK; 10 University of Southampton NHS Trust, London, UK; 11 Oxford University Hospitals NHS Foundation Trust, Oxford, UK; 12 Royal United Hospitals Bath NHS Foundation Trust, Bath, UK; 13 Department for Health, University of Bath, Bath, UK; 14 Imperial College Healthcare NHS Trust, London, UK; 15 University Health Network Toronto, Toronto General Hospital & Mount Sinai Hospital, University of Toronto, Toronto, ON Canada

**Keywords:** Pregnancy, Echocardiography, Obstetric

## Abstract

Pregnancy is a dynamic process associated with profound hormonally mediated haemodynamic changes which result in structural and functional adaptations in the cardiovascular system. An understanding of the myocardial adaptations is important for echocardiographers and clinicians undertaking or interpreting echocardiograms on pregnant and post-partum women. This guideline, on behalf of the British Society of Echocardiography and United Kingdom Maternal Cardiology Society, reviews the expected echocardiographic findings in normal pregnancy and in different cardiac disease states, as well as echocardiographic signs of decompensation. It aims to lay out a structure for echocardiographic scanning and surveillance during and after pregnancy as well as suggesting practical advice on scanning pregnant women.

## Introduction

Heart disease is the commonest cause of maternal death in the UK and other high income countries [1], and lessons can be learned that could have altered the outcome in more than half of cases. Most deaths have occurred in women who were not previously known to have had heart disease, and echocardiography is often the key tool in making a diagnosis in the acute setting. It is also crucial for assessing and risk stratifying women with known heart disease prior to and during pregnancy, providing a comprehensive assessment of cardiac anatomy, function and haemodynamics.

Pregnancy is a dynamic state that is associated with major hormonally mediated changes that result in numerous physiological adaptations. These comprise significant physiological and hemodynamic changes within the cardiovascular system, including structural and functional adaptations of the myocardium [2, 3]. These changes serve to meet the increased metabolic demands demanded of the mother, whilst supporting the growth and development of the fetus. The inability of the myocardium to adapt sufficiently to these changes during pregnancy and the puerperium may result in poor maternal and / or fetal outcomes [3]. It is important that echocardiographers and clinicians have an understanding of the normal and abnormal cardiac structural and functional adaptations seen both during pregnancy and the post-partum period.

This guideline, on behalf of the British Society of Echocardiography (BSE) and the United Kingdom Maternal Cardiology Society (UKMCS), aims to provide an overview of the following areas.

1. Normal echo findings during pregnancy.
  -Structural changes in normal pregnancy.
  -Functional changes during normal pregnancy.
2. Echocardiography of pregnancy induced heart disease.
  -Peripartum cardiomyopathy.
  -Pulmonary embolism.
  -Aortic dissection.
  -Acute coronary syndrome.
  -Hypertensive disorders of pregnancy.
3. Echocardiography in pregnancy in pre-existing heart disease
  -Valvular heart disease.
  -Cardiomyopathies.
  -Pulmonary arterial hypertension.
  -Congenital heart disease.
4. Echocardiography triage and surveillance during and after pregnancy.
5. Practical advice on scanning pregnant women.

Levels of evidence and strengths of recommendation are shown in Tables 1 and 2. These underscore the reality that a guideline does not take the place of high quality clinical surveillance and decision-making by the healthcare professionals involved in the care of the pregnant woman. The Class of Recommendation (Table 1) indicates the strength of the recommendation, incorporating the estimated benefit in proportion to risk. The Level of Evidence (Table 2) rates the quality of the scientific evidence supporting the recommendation, based on type, quantity, and consistency of data from clinical trials and other sources.

**Table 1:** Classes of recommendation

Classes of recommendations	Definition	Suggested wording to use
Class I	Evidence and/or general agreement that a given treatment or procedure is beneficial, useful, effective	Is recommended/is indicated
Class II	Conflicting evidence and/or a divergence of opinion about the usefulness/efficacy of the given treatment or procedure	
• Class IIa	Weight of evidence/opinion is in favour of usefulness/efficacy	Should be considered
• Class IIb	Usefulness/efficacy is less well established by evidence/opinion	May be considered
Class III	Evidence or general agreement that given treatment or procedure is not useful/effective and in some cases may be harmful	Is not recommended

**Table 2:** Levels of evidence

Level of evidence A	Data derived from multiple randomised clinical trials or meta-analyses
Level of evidence B	Data derived from a single randomised clinical trial or large non-randomised studies
Level of evidence C	Consenus of opinion of the experts and/or small studies, retrospective studies, registries

It should be noted that, whilst most studies should be done using the BSE Transthoracic Echocardiography (TTE) Minimum Dataset, on occasion a BSE Level 1 scan may be most appropriate, for example in acute admissions in the emergency department. Some frequently repeated studies in the cardiac antenatal clinic however will be focussed studies guided by senior clinicians, as a minimum dataset TTE will previously have been acquired. If not, a full study needs to be performed.

## Normal echo findings during pregnancy

### Structural changes in normal pregnancy

The most notable changes seen during pregnancy are an increase in blood volume, accommodated by an increase in heart rate and stroke volume, and consequently, cardiac output. These changes typically commence within the first trimester with an increase in cardiac output of up to 50% compared to non-pregnant levels [4]. The sustained increase in blood volume leads to balanced, subtle dilatation of all four cardiac chambers [5–7]. This begins at around 12 weeks of gestation and progresses throughout pregnancy [8]. However, it is important to note that during a normal pregnancy the extent of the dilatation is small with chamber sizes remaining within normal limits. Moreover, these changes recover within three to six months of delivery [6, 7, 9].

The increase in left ventricular dimensions results in eccentric symmetrical hypertrophy. There is a 5–10% overall increase in left ventricular mass [10] and wall thickness of 25–30% above pre-pregnancy levels, all staying within normal values [3]. LV sphericity progressively increases, occurring early, and recovering by three to six months post-partum [6, 9]. Hypertrabeculation has also been shown to develop, more commonly in Afro-Caribbean than Caucasian women, and this is thought to be due to increased preload, analogous to the changes that occur in the athlete’s heart [11]. These changes again resolve post-partum in the majority of women, though full resolution may take up to two years [11].

Asymptomatic small pericardial effusions of no haemodynamic consequence occur in approximately 40% of pregnancies. These are more common in the third trimester and if there has been a weight gain of > 12 kg in the pregnancy. These typically resolve by six weeks post-partum and do not need further review [12] (IIC).

### Functional changes during normal pregnancy

There is no significant change in left ventricular ejection fraction throughout pregnancy and post-partum [6, 9, 13–18]. However, a subtle decline to low normal [19] has been observed from the second trimester, persisting into the early post-partum period [5, 7].

Global longitudinal strain (GLS) steadily declines to the lower end of the normal range [5, 6, 14, 20] in the second trimester, after which it remains stable until term. An increase in GLS above normal is commonly seen post-partum [5, 6]. Data on circumferential and radial strain are sparse [6]. Left ventricular torsion increases during pregnancy, from the second trimester onwards until term, after which it returns to normal [14, 21].

Left ventricular diastolic function is more difficult to interpret during pregnancy as trans-mitral inflow is strongly influenced by loading conditions [8, 22, 23]. The increase in preload, coupled with a decrease in afterload in early pregnancy, results in an increase in mitral E wave velocity, a lowering of A wave velocity and corresponding increase in E/A ratio. These parameters return to normal within one year post-partum [18, 23]. E/eʹ, as a relatively load independent measure, remains unchanged throughout pregnancy [2, 5, 23–25]. The true incidence of diastolic dysfunction during pregnancy is unclear as many studies have used outdated methodologies [22] or non-standard definitions.

Data on right ventricular function in pregnancy are limited to a few small studies. GLS and fractional area change have been shown to decline to low normal as pregnancy progresses [26] but tissue Doppler imaging (TDI) peak Sʹ velocity has been shown not to change; all return to normal postpartum [26].

An illustration of normal intervals found in a study of 559 women is shown in Table 3. However, it should be noted that, in general, the subtle changes in parameters of ventricular function seen during pregnancy remain within normal limits. Echocardiographers should therefore still use the BSE guidelines to assess whether a chamber is enlarged.

**Table 3:** Typical changes in cardiac size (A) and function (B) of the left ventricle for 559 pregnant women by trimester adapted from [8]

	< 20 weeks	> 20 weeks
	Median	Median
A		
LVEDd (cm)	4.5	4.6
LVESd (cm)	3.1	3.1
LVEDvol (mL)	88	88
LVESvol (mL)	33	34
PWTd (cm)	0.8	0.8
IVSd (cm)	0.7	0.7
LA volume (mL)	54	54
LVMI (g/m^2^)	61	63
B		
LVEF (%)	61	61

Based on 137 women (< 20 weeks) and 274 women (20 + weeks) and shown as median with interquartile range

*LVEDd* left ventricular end diastolic dimension, *LVESd* left ventricular end systolic dimension, *LVEDvol* left ventricular end diastolic volume, *LVESvol* left ventricular end systolic volume, *PWTd* posterior wall thickness in diastole, *IVSd* interventricular septal wall thickness in diastole, *LVEF *left ventricular ejection fraction, *LA* left atrium, *LVMI* left ventricular mass index

Findings that should raise an alert and prompt clinical review can be found in Table 4. Care of pregnant is best jointly managed by a team of specialist cardiologists, obstetricians and obstetric anesthetists, known as the pregnancy heart team.

**Table 4:** Changes on echocardiography in pregnancy that should raise an alert and prompt clinical review [27, 40]

Condition		Level of evidence and strength of recommendation
General recommendations		
General	• Large one-off, or stepped changes in parameters, even if they remain in the normal range	IC
	• Measurements that fall outside normal values	IC
Arrhythmia	• Any abnormal rhythm, e.g. loss of sinus rhythm, frequent premature ventricular complexes	IC
Aortopathy	• Any progression of aortic dilatation	IC
Valvular heart disease		
Aortic stenosis	• Any decrease in LVEF, especially if accompanied by fall in transvalvar velocity	IC
	• Any increase in LV systolic or diastolic dimensions	IIC
	• Tachycardia on echo (> 100 beats per minute)	IC
Mitral stenosis	• Any new diagnosis of mitral stenosis • Progression of severity • New onset atrial fibrillation • Left atrial spontaneous contrast or suspicion of thrombus • Any deterioration in RV function, increase in PA pressure or increase in RV dimensions	IC IC IC IC IIC
Pulmonary stenosis	• Deterioration in RV function • Increasing severity of tricuspid regurgitation	IC IIC
Mitral regurgitation	• Deterioration in LV function • Increase in LV systolic or diastolic dimensions • Increase in severity of mitral regurgitation	IC IC IC
Aortic regurgitation	• Deterioration in LV function • Increase in LV systolic or diastolic dimensions • Increase in severity of aortic regurgitation	IC IC IC
Pulmonary regurgitation	• Progressive increase in RV dimensions • Reduction in RV systolic function • Increasing severity of tricuspid regurgitation	IIC IC IIC
Tricuspid regurgitation	• Progressive increase in RV dimensions • Reduction in RV systolic function • Increasing severity of tricuspid regurgitation	IIC IC IIC
Prosthetic valves	• Any suspicion of mechanical valve dysfunction suggesting possible valve thrombosis • Other imaging modalities (fluoroscopy and/or TOE, rarely CT^a^) should be considered	IC IIC
Cardiomyopathy		
PPCM	• New LV dysfunction	IC
	• Serial reduction in LV function	IC
	• Serial increase in LV dimensions	IC
	• Evidence of abnormalities associated with poor prognosis including:	
	• LVEF ≤ 30%	IC
	• LVEDd ≥ 6 cm	IC
	• RV dilatation and dysfunction	IIC
Dilated cardiomyopathy and previous PPCM	• Serial reduction in LV function • Serial increase in LV dimensions	IC IC
Hypertrophic cardiomyopathy	• Newly detected LVOT obstruction • Deterioration in systolic or diastolic LV function • Increase in E/e’ • Loss of sinus rhythm	IC IC IIC IIC
Arrhythmogenic cardiomyopathy	• Deterioration in ventricular function • Increase in degree of tricuspid regurgitation • Frequent or complex ventricular ectopy	IC IIC IIC
Pulmonary arterial hypertension	• Any deterioration in RV function • Evidence of rising pulmonary artery pressure • Progressive tricuspid regurgitation	IC IC IIC
Congenital heart disease		
Tetralogy of Fallot	• Deterioration in RV function • Progressive tricuspid regurgitation	IC IIC
Transposition of the great arteries (dTGA)	Post arterial switch operation:	
	• New LV dysfunction	IC
	• Progressive dilatation of neo aortic root or aortic regurgitation	IIC
	• If right-sided obstruction, worsening of RV function or progressive TR	IIC
	Post Senning or Mustard repair:	
	• Deterioration in systemic RV function	IC
	• Progression of systemic tricuspid regurgitation	IIC
	Post Rastelli operation:	
	• New LV dysfunction	IIC
Congenitally corrected TGA	• New or deteriorating systemic RV dysfunction • Progressive systemic tricuspid regurgitation	IC IIC
Fontan circulation	• New or deteriorating ventricular dysfunction • New or deteriorating atrioventricular valve regurgitation • Loss of sinus rhythm	IIC IIC IIC

Deterioration in ventricular function should be considered as a deterioration compared to the previous study. Any change in valve function should be considered as a change of ≥ 1 grade of stenosis or regurgitation

*DCM* dilated cardiomyopathy, *EF* ejection fraction, *LV* left ventricle/ventricular, *LVOT* left ventricular outflow tract, *LVEDd* left ventricular end diastolic diameter, *PA* pulmonary artery, *PPCM* peripartum cardiomyopathy, *RV *right ventricle/ventricular, *TGA* transposition of the great arteries, *TOE* transoesophageal echocardiography, *TR* tricuspid regurgitation

^a^CT should be used if fluoroscopy and/or TOE are not diagnostic. It should not be avoided because of the radiation dose in a potentially high risk situation

## Echocardiography of pregnancy induced heart disease

### Peripartum cardiomyopathy

Peripartum cardiomyopathy (PPCM) is defined as an idiopathic cardiomyopathy presenting with heart failure secondary to left ventricular systolic dysfunction towards the end of pregnancy or within the first five months following delivery, where no other cause of heart failure is found [27]. A reduction in left ventricular ejection fraction (usually < 45%) with or without left ventricular dilatation is required to establish a diagnosis. Around two thirds of women with PPCM develop it postpartum [27].

PPCM can be difficult to distinguish from pre-existing dilated cardiomyopathy (DCM) presenting de novo in pregnancy, although DCM may present at an earlier gestation. Echocardiographic assessment of PPCM and DCM is the same.

Echocardiography can help predict prognosis and /or recovery in PPCM [28]; a left ventricular ejection fraction of ≤ 30% at presentation confers a poorer prognosis and reduced likelihood of recovery [28], as does a global longitudinal strain of > -10.6%, global circumferential strain of < 10.1% [29], and left ventricular end diastolic diameter (LVEDd) of ≥ 6 cm [28]. Impaired right ventricular (RV) function co-exists in a quarter to a third of women with PPCM [30–32]. Again, echocardiographic measurements can be indicative of a poorer prognosis: TAPSE (tricuspid annular plane systolic excursion) < 16 mm, TDI peak Sʹ velocity of < 10 cm/s [32], fractional area change < 36%, and right ventricular end systolic area > 13 cm^2^ [33].

Echocardiography is also important in the assessment of left ventricular thrombus, which may complicate significant left ventricular impairment due to the hypercoagulable state of pregnancy [34].

There are no safety data for echo contrast agents in pregnancy and they should therefore generally be avoided unless the maternal benefit is considered to outweigh the risk. This decision can be made by a senior physician from the pregnancy heart team.

### Pulmonary embolism

Pulmonary embolism (PE) is the most common “direct” (i.e. not pre-existing but relating to pregnancy) cause of maternal death in the UK [35]. Acute PE may lead to characteristic changes on echo, including reduced pulmonary acceleration time, reduced longitudinal motion of the free wall compared to a relatively hyperdynamic RV apex, signs of RV pressure overload, such as systolic septal flattening in the PSAX view, and thrombus in the right heart. While echocardiography is not the primary imaging modality to diagnose or exclude PE, it may point to alternative diagnoses and is of value in risk stratifying a proven PE [36].

### Aortic dissection

Whilst aortic dissection is uncommon during pregnancy, it accounts for 11% of maternal cardiac deaths in the UK [1], mostly occurring in the third trimester or early post-partum. Pregnancy increases the risk of dissection due to the hormonal and haemodynamic effects on the aortic wall [37, 38] and most affected women do not have a prior diagnosis of aortopathy [1]. It is often the dissection that leads to the underlying diagnosis, e.g. as Marfan, Loeys-Dietz and Turner syndromes [1, 38].

Patients with aortopathy are considered to be at highest risk if the aorta is > 45 mm in hereditary aortopathies, > 50 mm in bicuspid aortic valve associated aortopathy and > 25 mm/m^2^ in Turner syndrome. Identifying progressive dilatation in patients with known aortopathy is particularly important and these women should have regular echocardiographic surveillance. Images should be carefully compared with previous studies [39]. Recommended surveillance intervals are shown in Table 6.

Patients with a repaired or replaced aortic root remain at risk of more distal dilatation or dissection. Thus, echocardiography should include suprasternal and subcostal views to ensure that as many parts of the aorta are imaged as possible. Imaging with non-contrast magnetic resonance imaging to assess the entire aorta is safe during pregnancy and does not require the use of gadolinium contrast.

Any progression of aortic dilatation in a pregnant patient with known aortopathy should raise an alert and prompt clinical review [40].

### Acute coronary syndrome

Acute coronary syndromes in pregnancy may be due to atherosclerosis, pregnancy-related spontaneous coronary artery dissection or acute intra-coronary thrombosis [40]. Echocardiographic assessment is as for non-pregnant patients.

### Hypertensive disorders of pregnancy

Hypertensive disorders of pregnancy are common, affecting around 10% of pregnancies [40, 41]. The term “Hypertensive disorders of pregnancy”, (HDP) includes chronic hypertension in pregnancy, gestational hypertension and preeclampsia [42]. These conditions are important as they are associated with a two-fold increase in the risk of longer-term cardiovascular disease [44, 45] and a sixfold increase in the risk of developing hypertension within two years of delivery [46]. In general, the BSE only recommends echocardiography if there is a clinical suspicion of heart failure or coarctation of the aorta (IIC) [43].

If echocardiography is performed, the focus should be on the assessment of concentric hypertrophy, left atrial dilatation [47] and diastolic dysfunction [48]. Left ventricular ejection fraction does not appear to be affected by gestational hypertension [48, 49], although global longitudinal strain has been shown to be reduced [50].

## Echocardiography in pregnancy in pre-existing heart disease

### Valvular heart disease

#### Normal findings in pregnancy

The haemodynamic changes of pregnancy influence the maternal response to and echocardiographic assessment of valve disease. Due to chamber dilatation, the mitral, and tricuspid annuli increase during pregnancy, resulting in mild mitral and tricuspid regurgitation in 28% and 94% cases, respectively. The pulmonary annulus also dilates, resulting in mild pulmonary regurgitation in 94% of cases. These changes have mostly resolved by six weeks post-partum but can take up to six months to fully resolve [51]. The aortic root diameter increases a small amount but remains within normal limits. The annulus does not change, and so aortic regurgitation is not a feature of normal pregnancy [51].

Transvalvular gradients increase throughout pregnancy [6, 52, 53], reflecting the increasing stroke volume [6]. However, it should be remembered the standard cut off values to determine severity of valve stenosis are based on normal flow rates outside of pregnancy. Velocity-derived pressure gradients correlate less well with stenosis severity, though valve area calculations using the Bernoulli equation and assessment of the dimensionless valve index (DVI) are still valid [52, 53]. Assessment of stenosis using the modified Bernoulli equation alone can overestimate the stenosis and should be avoided, due to the increased flow state. Indexing of AVA to current weight derived BSA should also be avoided.

In general, due to the reduction in systemic vascular resistance, regurgitant valve lesions are better tolerated in pregnancy than stenotic ones, and are associated with better outcomes [54, 55].

Assessment of valve disease should follow previously published BSE guidance [56–58]. However, there are some echocardiographic parameters that are particularly important in assessing valvular heart disease during pregnancy. These are detailed below and shown in Table 5.

**Table 5:** Key measurements for the serial echocardiographic assessment of valves in pregnancy, which most affect decision making (see text)

	Left heart measurements	Right heart measurements
Mitral stenosis	Mean gradient	Estimation of PAP
Mitral regurgitation	Degree of MR LV size and function	Estimation of PAP
Aortic stenosis	LV function	Estimation of PAP
Aortic regurgitation	LV size and function Note if aortic root dilation is cause	Estimation of PAP
Tricuspid regurgitation		Degree of TR RV size and function
Pulmonary stenosis		RV size function
Pulmonary regurgitation		RV size and function Degree of TR Estimation of PAP if PH

*LV* left ventricle, *PAP* pulmonary artery pressure, *PH* pulmonary hypertension, *RV* right ventricle, *TR* tricuspid regurgitation, *MR* mitral regurgitation

### Aortic stenosis

Aortic stenosis in women of childbearing age in high income countries is most often related to bicuspid aortic valve disease, either operated or unoperated. Rheumatic heart disease is more common in women from low and middle income countries. Echocardiography is key in the assessment of the ability of the left ventricle to cope as cardiac output increases. Systolic and diastolic function, filling pressure, left atrial size, mitral regurgitation and pulmonary artery pressure should be assessed. In those with bicuspid aortic valve disease and/or previously identified aortic dilatation, the aortic root and ascending aorta should be measured [59].

### Mitral stenosis

Mitral stenosis in women of childbearing age is most commonly due to rheumatic heart disease [54] but can also be seen in parachute mitral valve, or after mitral valve repair (or atrioventricular valve repair in the context of atrioventricular septal defect). Rheumatic mitral stenosis remains prevalent in patients born in low and middle income countries, and not uncommonly presents for the first time late in the second trimester, when the cardiac output starts to peak and even women with moderate mitral stenosis can decompensate in pregnancy.

Careful assessment is needed of the underlying cause of mitral stenosis, the severity, and other associated valvar or structural lesions. Reporting the mean gradient (and heart rate) is most helpful, acknowledging that the gradient will increase as pregnancy progresses. In cases of newly diagnosed mitral stenosis in pregnancy, sole use of the mean gradient can overestimate the degree of stenosis. Assessment of effective valve orifice area by planimetry (if easily obtained) or continuity equation is preferred to the pressure half time method which is less reliable in pregnancy. Echocardiographic assessment should focus on left ventricular filling pressure, left atrial size and thrombus, pulmonary artery pressure, and right heart size and function.

The morphology of the stenotic mitral valve should be carefully assessed, including feasibility of balloon mitral valvuloplasty, since it is usually only the rheumatic valve that is suitable for percutaneous valvuloplasty.

### Pulmonary stenosis

Pulmonary stenosis in pregnancy is rare. Most cases are congenital and have been treated in childhood. Where seen it is generally well tolerated as long as RV function is maintained [60]. Echocardiographic assessment should focus on RV systolic function. If impairment is present, surveillance may be required. If decompensation occurs in severe pulmonary stenosis (peak gradient > 64 mmHg) during pregnancy, balloon pulmonary valvuloplasty may be indicated.

### Mitral regurgitation

Most mitral regurgitation in women of childbearing age is either due to mitral valve prolapse, secondary to annular dilation, or rheumatic heart disease [55, 61]. The benefit of the reduction in afterload in mitral regurgitation is offset by the expansion in blood volume, which together with chamber and annular dilation results in an increased regurgitant volume. Echocardiographic focus should be on left ventricular size, systolic and diastolic function, filling pressure, left atrial size, progression of mitral regurgitation and estimation of PA pressure.

### Aortic regurgitation

Aortic regurgitation in women of childbearing age is most commonly due to bicuspid aortic valve disease, including those with previous valvotomy or balloon valvuloplasty. Rare causes include aortopathy-related annular dilation, cusp prolapse, previous endocarditis, or rheumatic valve disease. In aortic regurgitation the reduction in systemic vascular resistance and increased heart rate of pregnancy reduces the effective regurgitant volume. It is rarely problematic and maternal and fetal risks are low [55, 61]. Echocardiographic focus should be on left ventricular size, systolic and diastolic function, filling pressure, progression of aorta size and/or aortic regurgitation and estimation of PA pressure.

### Pulmonary regurgitation

In pregnancy, pulmonary regurgitation is usually encountered in women with known congenital heart disease due to repaired Tetralogy of Fallot, pulmonary atresia or previous pulmonary valvotomy/valvuloplasty for congenital pulmonary stenosis. It is well tolerated if the right ventricular function is good [61, 62]. Rarely it may be secondary to pulmonary arterial hypertension, which carries a very high maternal risk. If the right ventricle dilates, tricuspid regurgitation may develop or worsen. Echocardiographic focus should be on diagnosing the underlying aetiology, right ventricular size and systolic function and degree of tricuspid regurgitation.

### Tricuspid regurgitation

In women of child-bearing age, tricuspid regurgitation may be due to congenital heart disease, such as Ebstein’s anomaly, or secondary to an unrepaired atrial septal defect or pulmonary regurgitation, when the right ventricle and tricuspid annulus are dilated. It may also be acquired, as a post intervention phenomenon (post-surgical repair of ventricular septal defect (VSD), or due to a transvalvar pacing lead), or due to previous endocarditis or trauma. Primary tricuspid regurgitation often progresses in pregnancy due to the volume load and annular dilatation. Echocardiographic focus should be on right ventricular size, systolic function, progression of regurgitation and any associated lesion [61].

### Prosthetic valves

Forward flow velocities are marginally increased across all prosthetic valves in pregnancy, in keeping with the increase in cardiac output. The mean gradient is less flow-dependent and serial assessment of DVI is useful. An abrupt increase in velocity mandates careful assessment in the context of symptoms and new clinical findings. Encountering a high gradient secondary to patient prosthesis mismatch (PPM) de novo in pregnancy, without recourse to any previous post-operative echo, is an uncommon scenario. Leaflet motion is normal in PPM and aortic valve acceleration time will not be significantly prolonged. Quantification of prosthetic valve related regurgitation mirrors assessment for native valves.

Pregnancy in a woman with a tissue valve replacement is well tolerated if left ventricular systolic function is within normal limits and there is no/minimal prosthetic valve dysfunction. Management is similar to that of native valve disease and the prosthetic valve should be assessed in the same way as for the non-pregnant patient.

Pregnancy in a woman with a mechanical valve replacement is associated with significant fetal and maternal morbidity and mortality [63, 64]. Prosthetic valve thrombosis can occur, particularly if a woman chooses to use low molecular weight heparin instead of warfarin (which crosses the placenta and is teratogenic). Frequent (four weekly) echocardiographic surveillance of the valve is important, regardless of the anticoagulation regime. Sudden breathlessness or presentation with a possible thromboembolic event during pregnancy or up to six weeks after delivery mandates same-day echocardiography for careful interrogation of the prosthesis [40].

In patients with prosthetic valves and symptoms suggestive of valve dysfunction, or evidence of an abrupt increase in transvalvar velocities or worsening regurgitation, meticulous assessment of the opening and closing of the valve leaflets for evidence of thrombus (or a vegetation) should be sought. If valve leaflet dysfunction is suspected, other imaging modalities are likely to be needed (fluoroscopy and/or trans-oesophageal echocardiography [TOE], rarely CT). Any suspicion of mechanical valve dysfunction should raise the possibility of valve thrombosis, and trigger urgent clinical review (1C).

### Cardiomyopathies

Pre-existing cardiomyopathy in pregnancy may be dilated, arrhythmogenic, hypertrophic or previous peripartum. Each is discussed briefly below.

### Dilated cardiomyopathy

Dilated cardiomyopathy (DCM) seen in pregnancy may be familial or acquired, for example due to previous myocarditis, previous chemotherapy, but also hypertension, diabetes or alcohol. In many cases, the cause may be unknown. Although many will have been diagnosed prior to pregnancy and thus had the opportunity for pre-pregnancy assessment and counselling, some women do not present until the physiological demands of pregnancy precipitate symptoms for the first time.

Many pregnant women with known DCM have a left ventricular ejection fraction > 40%, as those with more severe dysfunction (LVEF < 30%) [40] are usually counselled against, and may choose not to become pregnant. Nonetheless, pregnancy can precipitate heart failure with a reduction in ventricular function. Left ventricular systolic function must be assessed serially for a decline in function. Global longitudinal strain may be useful in identifying women at higher risk of deterioration, but data are sparse on normal and abnormal cut off values. Echocardiography assessment should focus upon the DCM assessment as per non-pregnant patients [65].

### Hypertrophic cardiomyopathy

Pregnancy in women with hypertrophic cardiomyopathy is well tolerated if left ventricular systolic and diastolic function are within normal limits and there is no significant left ventricular outflow tract (LVOT) obstruction [66, 67]. In women at higher risk, the increased load of pregnancy can result in decompensation, as can the onset of atrial arrhythmia. Echocardiographic focus is as per previously described guidance [47] and should include left ventricular systolic and diastolic function, assessment for left ventricular outflow tract (LVOT) obstruction and serial assessment of E/e’ [68].

### Arrhythmogenic cardiomyopathy

Arrhythmogenic cardiomyopathy primarily affects the right ventricle and is rare. Heart failure in pregnancy can occur in women with previous ventricular impairment [69]. Focus should be on left and right ventricular systolic function, as the left ventricle is frequently involved.

### Previous peripartum cardiomyopathy

Approximately half of women who suffer from PPCM will recover [70]. Some may choose to have another pregnancy and, in those that do, the recurrence rate is approximately 25%. This figure rises to two thirds in those who have residual left ventricular systolic dysfunction (ejection fraction < 45%) [71]. Echocardiographic assessment is as for DCM above [65].

### Pulmonary arterial hypertension

Pulmonary arterial hypertension (PAH) carries an extremely high maternal risk, when due to elevated pulmonary vascular resistance, even in the era of selective pulmonary vasodilator therapy. Echocardiographic assessment should form part of specialised, frequent clinical assessment throughout pregnancy and the early puerperium, and focus on surveillance of right ventricular size, function and estimated pulmonary artery pressure [40].

### Congenital heart disease

Understanding the anatomy and physiology of the congenital lesion, details of any repair and the effects of the physiological changes of pregnancy on this are essential [72]. The BSE and UKMCS advocate that pregnant women with complex lesions should be scanned by appropriately trained and experienced echocardiographers.

### Septal defects and left to right shunts

Isolated small or repaired septal defects and left to right shunts with no significant sequelae, including partial anomalous pulmonary venous drainage, atrial and ventricular septal defects and patent arterial ducts do not usually require echocardiographic surveillance during pregnancy. Unrepaired larger atrial septal defects are well tolerated in pregnancy if right ventricular function and pulmonary vascular resistance are normal. The increased volume load of pregnancy will result in an increase in left to right shunting, but this is ameliorated by a reduction in systemic vascular resistance. Tricuspid regurgitation may develop and should be assessed accordingly. Atrioventricular septal defects will usually have been repaired prior to pregnancy. Any residual left atrioventricular valve disease should be approached as per the mitral valve and any residual atrial septal defect as an unrepaired atrial septal defect.

### Tetralogy of fallot

Most patients will have undergone surgical repair and may have had a further pulmonary valve replacement for pulmonary regurgitation. They may also have had intervention to the branch pulmonary arteries and/or tricuspid valve. Patients with native pulmonary valves in situ not uncommonly have chronic severe pulmonary regurgitation. Echocardiographic focus should be on assessing right ventricular function, preferably using strain, and comparing serial measurements [73]. Pulmonary regurgitation does not usually increase significantly but tricuspid regurgitation is common and may cause symptoms if severe. Pregnancy is usually well tolerated if right ventricular function is good [74].

### Transposition of the great arteries (dTGA)

Most patients undergoing surgical correction from the 1990s onwards will have had an arterial switch repair, which involves a switch at great artery level with re-implantation of the coronary buttons. Post-operatively, the cardiac chambers are in their usual positions. Complications, such as supra-pulmonary stenosis, neo-aortic root dilatation, aortic regurgitation and left ventricular dysfunction, should be assessed in the same way as in the non-pregnant patient.

Pre-1990s, surgery for TGA involved rerouting systemic and pulmonary venous return at atrial level, with a Mustard or Senning repair, resulting in a systemic morphological right ventricle, systemic tricuspid valve, and a subpulmonary left ventricle. Late complications include systemic right ventricular dysfunction, systemic tricuspid regurgitation, and arrhythmias. Pregnancy can be associated with clinically significant progressive right ventricular dilatation and dysfunction, as well as an increase in systemic tricuspid regurgitation [75].

A Rastelli repair may be performed to repair transposition of the great arteries with VSD and pulmonary stenosis. The VSD is closed to commit the left ventricle to the aorta, the pulmonary artery is ligated proximally, and a right ventricle to pulmonary artery conduit is placed, resulting in a systemic morphological left ventricle. Assuming good ventricular and conduit function, pregnancy is generally well tolerated, with slightly increased forward flow through the conduit as the cardiac output rises.

### Congenitally corrected TGA

In congenitally corrected TGA (ccTGA) the morphological right ventricle is the systemic ventricle and there is a subpulmonary left ventricle. Progressive systemic right ventricular dilatation and dysfunction compared to baseline may occur with pregnancy. An increase in systemic (tricuspid) valve regurgitation may also be noted.

### Fontan circulation

A Fontan circulation is created to palliate the functionally single ventricle heart. The single ventricle supports the systemic circulation, with passive flow to the pulmonary arteries. The efficiency of this limited cardiac output circulation is dependent on adequate filling, and maintenance of sinus rhythm. Pregnancy is associated with an increased risk of heart failure, arrhythmia, haemorrhage and thrombosis [76]. Echocardiographic assessment should focus on detecting any progression of ventricular dysfunction and/or valvar regurgitation.

## Echocardiography triage and surveillance during and after pregnancy

Echocardiography triage and surveillance recommendations for pregnancy are shown in Table 6. Guidance in the literature is somewhat limited for many lesions and so we present our consensus view, based on experience and the likelihood of deterioration during pregnancy. The only conditions where first trimester screening is necessary are in patients presenting de novo, and those who are at very high risk, e.g. those with severe left ventricular systolic impairment, severe left ventricular outflow tract obstruction with symptoms, mechanical prosthetic valves, and high risk aortopathies. In higher risk women, echocardiographic surveillance needs to increase in frequency as pregnancy progresses. Monitoring frequency should increase if cardiac symptoms worsen, the clinical scenario changes or if echocardiographic parameters deteriorate. Conditions by risk, according to the European Society of Cardiology guidelines, are shown in Table 7 [40].

**Table 6:** Suggested frequency of echocardiography surveillance during and after pregnancy [27, 39, 40]

Condition	During pregnancy	Post-partum	Level of evidence and strength of recommendation
Valvular disorders^a^	As early as possible if not performed pre-pregnancy		
Stenotic lesions	Mild AS:		
	Once mid gestation only	3–5 years	IIaC
	Mild MS:		
	Once 2nd and 3rd trimester	3–5 years	IIaC
	Moderate AS:		
	Once 2nd and 3rd trimester and prior to delivery	12 months	IIaC
	Severe AS:		
	4–8 weekly depending on symptoms, and prior to delivery	Pre-discharge, then 3 months	IC
	Moderate and severe MS:		
	4–8 weekly depending on symptoms, and prior to delivery	Pre-discharge, then 3 months	IC
Regurgitant lesions	Mild AR/MR, moderate PR/TR:		
	Once mid gestation	As per usual follow-up	IIaC
	Moderate AR/MR, severe PR/TR:		
	Once 2nd and 3rd trimester	6 months	IIaC
Tissue prostheses	Severe: S Severe AR/MR:		
	8 weekly depending on symptoms	Pre-discharge, then 6 months	IIaC
Mechanical prostheses	As for native valve disease	Pre-discharge, then 1–2 months	IC
	4 weekly if functionally normal		IC
Cardiomyopathy			
Dilated cardiomyopathy	4–8 weekly, increasing to 1–4 weekly, if LVEF <40%	Pre-discharge, then 4–8 weeks, depending on ventricular function/recovery	IC
Hypertrophic cardiomyopathy	If symptomatic, systolic dysfunction, impaired diastolic function with raised filling pressure or obstruction, 4 weekly, otherwise once only	Pre-discharge, then 4-8 weeks if complicated or obstruction, otherwise 3–6 months	IIaC
ARVC	4–8 weekly, depending on RV function	Pre-discharge, then 4 weeks, if impaired RV function, otherwise 3–6 months	IIaC
Previous peripartum cardiomyopathy , with recovered LV function	End first trimester and end second trimester	Prior to discharge and one month after delivery	IC
Inherited aortopathies	4–12 weekly, depending on clinical scenario, as determined by the pregnancy heart team^b^	Pre-discharge, then 4–12 weeks, depending on clinical scenario	IC
Congenital heart disease^c^			
ASD with right heart dilatation, unrepaired	Once, not required if small	As per usual follow-up	IIaC
Fallot	If impaired RV function 4–8 weekly, otherwise once	Pre-discharge and 4–8 weeks if impaired RV function, otherwise 3–6 months	IIaC
TGA with systemic right ventricle	4–8 weekly, depending on clinical scenario	Pre-discharge, then 4–8 weeks	IIaC
Fontan	8 weekly, depending on clinical scenario/once	Pre-discharge only impaired ventricular function, otherwise 3–6 months	IIaC
Coronary artery disease	Once only, if no residual ventricular dysfunction	As per usual follow up	IIC
Hypertensive disorders of pregnancy	Only if suspicion of heart failure, or coarctation of the aorta	Not usually indicated	IC
Pulmonary arterial hypertension	4 weekly, increasing to 2–4 weekly, depending on RV function, as determined by the pregnancy heart team	Prior to discharge and at 4–8 weeks	IC

*ARVC *arrhythmogenic right ventricular cardiomyopathy, *AR* aortic regurgitation, *AS* aortic stenosis, *ASD *atrial septal defect, *EF* ejection fraction, *LV* left ventricular, *MR* mitral regurgitation, *MS* mitral stenosis, *PR* pulmonary regurgitation, *RV* right ventricular, *TGA* transposition of the great arteries, *TR* tricuspid regurgitation

^a^Degree of valve stenosis/regurgitation is based on pre-pregnancy scan. Post-partum follow up of mild and moderate valve disease can, in general, be as per BSE guidelines. In more severe disease, the pregnancy or simply the passage of time may have progressed the disease and earlier follow up is advised

^b^Frequency depends on the risk of dilatation, which is related to the aortic diameter, the underlying condition and the family history of dissection

^c^Dictated by ventricular function and valve disease

**Table 7:** Modified World Health Organization (WHO) classification of maternal cardiovascular risk with maternal cardiac event rate

mWHO I (no risk)	mWHO II (low risk	mWHO II-III (moderate risk)	mWHO III (high risk)	mWHO IV (pregnancy contraindicated)
Mild		Native/tissue valve disease not WHO I or IV	Mechanical valve	Severe MS Severe symptomatic AS
-Pulmonary stenosis			Moderate MS	
-Mitral valve prolapse			Severe asymptomatic AS	
Repaired simple shunts	Unoperated simple shunts	Mild LV impairment (EF > 45%) HCM	Moderate LV impairment (EF 30–45%) Systemic RV, good/mildly decreased function Recovered PPCM	Severe systemic ventricular dysfunction (EF < 30%/NYHA class III–IV) or > / = moderate if systemic RV PPCM, not recovered
PDA	Repaired Tetralogy of Fallot	AVSD Repaired coarctation	Fontan Unrepaired cyanotic heart disease/complex heart disease	Fontan with complication Severe (re)coarctation
Atrial or ventricular ectopic beats	Supraventricular arrhythmias		VT	
	Turner syndrome, no aortic dilatation	Marfan/HTA, no aortic dilatation Bicuspid aortopathy < 45 mm	Moderate aortic dilatation	Severe aortic dilatation Vascular Ehlers–Danlos
				PH
2.5–5%	5.7–10.5%	10–19%	19–27%	40–100%

*AS *aortic stenosis, *AVSD* atrioventricular septal defect, *EF* ejection fraction, *HCM* hypertrophic cardiomyopathy, *LV* left ventricle, *MS* mitral stenosis, *PDA* patent ductus arteriosus, *PH* pulmonary hypertension, *PPCM* peripartum cardiomyopathy, *RV* right ventricle, *VT* ventricular tachycardia

Adapted from [40]

**Fig. 1:**
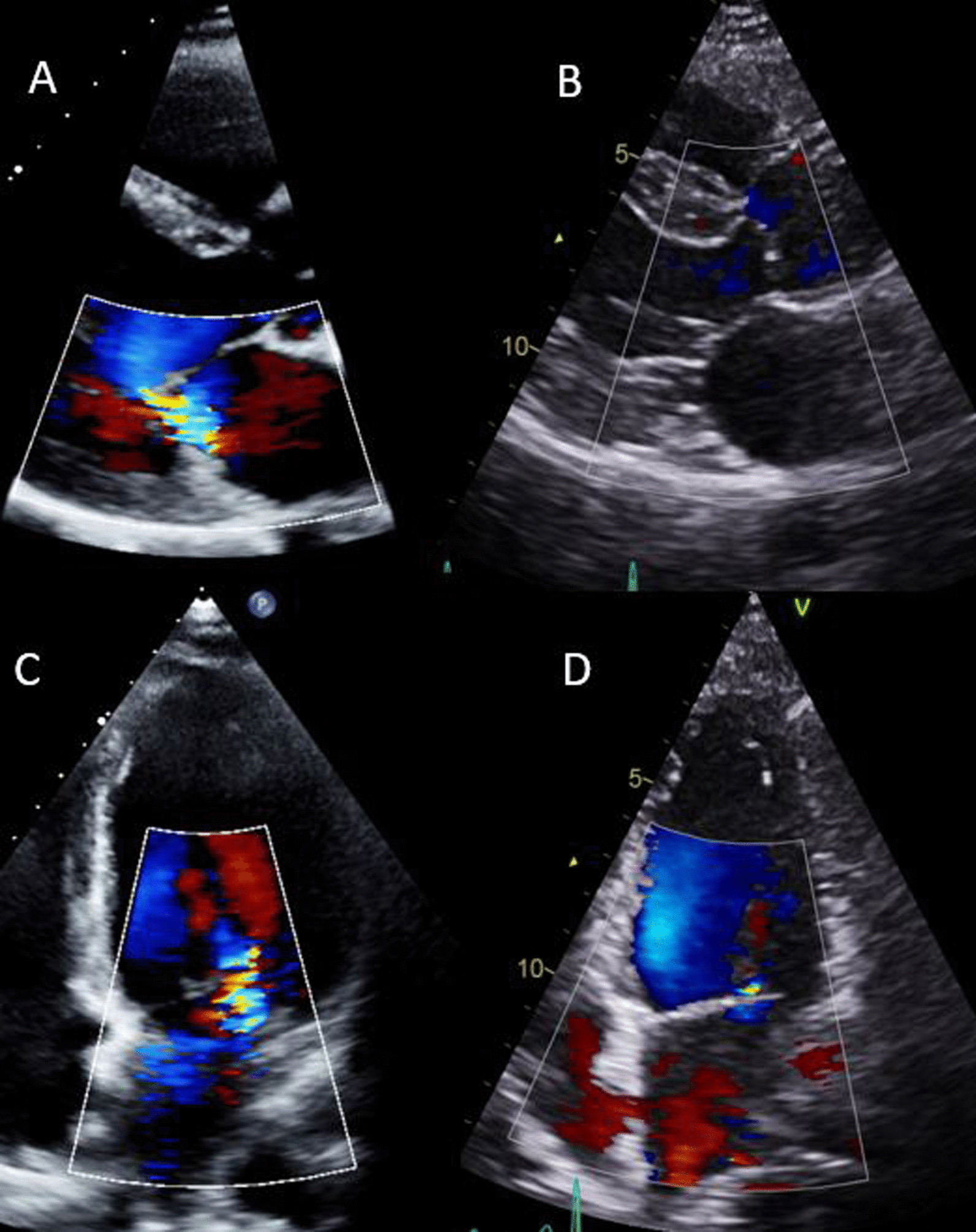
Post-partum hypertension on a background of mild anterior mitral valve leaflet prolapse with trivial mitral regurgitation, leading to severe mitral regurgitation and pulmonary oedema in a woman 24 h after delivery. **A** and **C** show the post-partum images. **B** and **D** show the same patient 6 months later on no medication

Though the symptoms and signs of cardiac decompensation are similar to the symptoms and clinical findings of normal pregnancy, breathlessness out of proportion to gestation, orthopnoea, paroxysmal nocturnal dyspnoea, resting sinus tachycardia > 100 beats per minute, and clinical signs of heart failure, all warrant prompt cardiology assessment and consideration of echocardiography. In addition, N-terminal pro hormone brain natriuretic peptide (NT pro-BNP) levels are only slightly elevated in normal pregnancy, more so in pre-eclampsia and markedly so in heart failure, including PPCM [77]. Therefore echocardiography should be undertaken as a clinical priority in pregnant women with symptoms or signs of heart failure, an abnormal 12 lead electrocardiogram or an NT pro-BNP in the heart failure range. It is important to note that echocardiography cannot exclude PE, acute coronary syndrome or aortic dissection, and other imaging modalities should be sought. Rapid increases in afterload due to hypertension, particularly associated with large fluid shifts such as those that occur post-partum, can result in worsening mitral regurgitation that can become clinically significant, as shown in Fig. 1.

### Practical advice on scanning pregnant women

Some special considerations are required for performing echocardiography in pregnant women. As the uterus and breasts increase in size as pregnancy advances, the ability to acquire high quality images decreases. The use of breath-hold will help improve image acquisition. The subcostal view becomes very difficult with advancing pregnancy. A patient > 20 weeks gestation should not be placed in the supine position, to avoid caval compression by the gravid uterus. Not only will this affect measurements dependent on cardiac output, but the patient may become hypotensive and syncopal.

Moving into the appropriate position for scanning may be difficult for the woman and breast tissue may be tender. It is therefore important to ensure that the patient is comfortable and feels at ease with the echocardiographer. It is helpful to take every measurement possible and assess lesions from multiple scanning views while image quality is good as the quality may deteriorate later in the pregnancy. Serial scans are often needed and so it is helpful to have scans performed by the same echocardiographer wherever possible to reduce interobserver variability.

## Conclusion

In response to the altered loading conditions of pregnancy, the normal heart undergoes reversible remodelling that may take weeks or months to resolve post-partum. These pregnancy-induced cardiovascular changes can stress maternal physiology sufficiently to adversely affect women with pre-existing cardiac disease. Pregnancy can also unmask previously undiagnosed cardiac conditions, or precipitate de novo disease. Sonographers and clinicians should understand the normal echocardiographic findings in pregnancy and recognise the abnormal changes that should precipitate prompt clinical review. As a safe, easily accessible imaging modality, echocardiography is crucial for the rapid diagnosis and surveillance of maternal heart disease, playing a key role in improving maternal outcomes.

## Data Availability

Not applicable.

## Abbreviations

ARVC: Arrhythmogenic right ventricular cardiomyopathy
ASD: Atrial septal defect
AS: Aortic stenosis
AV: Atrioventricular
AVSD: Atrioventricular septal defect
BSE: British Society of Echocardiography
ccTGA: Congenitally corrected transposition of the great arteries
DCM: Dilated cardiomyopathy
EF: Ejection fraction
GLS: Global longitudinal strain
HCM: Hypertrophic cardiomyopathy
HDP: Hypertensive disorders of pregnancy
IVSd: Interventricular septal wall thickness in diastole
LA: Left atrium
LV: Left ventricle
LVEDd: Left ventricular end diastolic dimension
LVEDvol: Left ventricular end diastolic volume
LVESd: Left ventricular end systolic dimension
LVESvol: Left ventricular end systolic volume
LVEF: Left ventricular ejection fraction
LVMI: Left ventricular mass index
LVOT: Left ventricular outflow tract
MR: Mitral regurgitation
MS: Mitral stenosis
NT pro-BNP: N-terminal pro hormone brain natriuretic peptide
PAP: Pulmonary artery pressure
PDA: Patent ductus arteriosus
PH: Pulmonary hypertension
PPCM: Peripartum cardiomyopathy
PE: Pulmonary embolism
PWTd: Posterior wall thickness in diastole
RV: Right ventricular/right ventricle
TASPE: Tricuspid annular plane systolic excursion
TDI: Tissue Doppler imaging
TGA: Transposition of the great arteries
TR: Tricuspid regurgitation
TOE: Trans-oesophageal echocardiography
TTE: Trans-thoracic echocardiography
UKMCS: United Kingdom’s Maternal Cardiology Society
VT: Ventricular tachycardia
WHO: World Health Organisation
